# A qualitative study using traditional community assemblies to investigate community perspectives on informed consent and research participation in western Kenya

**DOI:** 10.1186/1472-6939-13-23

**Published:** 2012-09-25

**Authors:** Rachel Vreeman, Eunice Kamaara, Allan Kamanda, David Ayuku, Winstone Nyandiko, Lukoye Atwoli, Samuel Ayaya, Peter Gisore, Michael Scanlon, Paula Braitstein

**Affiliations:** 1 Department of Pediatrics, Indiana University School of Medicine, 705 Riley Hospital Drive, Room 5900, Indianapolis, IN, 46202, USA; 2 USAID - Academic Model Providing Access to Healthcare (AMPATH) Partnership, P.O. Box 4606, Eldoret, 30100, Kenya; 3 Regenstrief Institute, Inc., 410 W. 10th St., Suite 2000, Indianapolis, IN, 46202-3012, USA; 4 Departments of Philosophy and Religious Studies, Moi University, P.O. Box 3990, Eldoret, 30100, Kenya; 5 Moi Teaching and Referral Hospital, Nandi Road, P.O. Box 3, Eldoret, 30100, Kenya; 6 Department of Behavioural Sciences, School of Medicine, Moi University College of Health Sciences, P.O. Box 4606, Eldoret, 30100, Kenya; 7 Department of Child Health and Paediatrics, School of Medicine, Moi University College of Health Sciences, P.O. Box 4606, Eldoret, 30100, Kenya; 8 Department of Mental Health, School of Medicine, Moi University College of Health Sciences, P.O. Box 4606, Eldoret, 30100, Kenya; 9 Department of Medicine, Indiana University School of Medicine, 545 Barnhill Drive, EH 317, Indianapolis, IN, 46202, USA; 10 Children’s Health Services Research, Health Information and Translational Sciences Building, Suite #1000, 410 W. 10th St., Indianapolis, IN, 46202, USA

**Keywords:** Community-based research, Sub-Saharan Africa, Ethics, Informed consent, Kenya

## Abstract

**Background:**

International collaborators face challenges in the design and implementation of ethical biomedical research. Evaluating community understanding of research and processes like informed consent may enable researchers to better protect research participants in a particular setting; however, there exist few studies examining community perspectives in health research, particularly in resource-limited settings, or strategies for engaging the community in research processes. Our goal was to inform ethical research practice in a biomedical research setting in western Kenya and similar resource-limited settings.

**Methods:**

We sought to use *mabaraza*, traditional East African community assemblies, in a qualitative study to understand community perspectives on biomedical research and informed consent within a collaborative, multinational research network in western Kenya. Analyses included manual, progressive coding of transcripts from *mabaraza* to identify emerging central concepts.

**Results:**

Our findings from two *mabaraza* with 108 community members revealed that, while participants understood some principles of biomedical research, they emphasized perceived benefits from participation in research over potential risks. Many community members equated health research with HIV testing or care, which may be explained in part by the setting of this particular study. In addition to valuing informed consent as understanding and accepting a role in research activities, participants endorsed an increased role for the community in making decisions about research participation, especially in the case of children, through a process of community consent.

**Conclusions:**

Our study suggests that international biomedical research must account for community understanding of research and informed consent, particularly when involving children. Moreover, traditional community forums, such as *mabaraza* in East Africa, can be used effectively to gather these data and may serve as a forum to further engage communities in community consent and other aspects of research.

## Background

To prevent exploitation of human subjects and build true collaborative research partnerships with local communities, researchers conducting biomedical or behavioral research in resource-limited settings must consider the plethora of ethical challenges particular to the culture(s) and society in which the work will occur [1-4]. While there are overarching ethical guidelines for biomedical and behavioral research, set forth in documents like the Declaration of Helsinki and by ethics research bodies like the Council for International Organizations of Medical Sciences and Nuffield Council on Bioethics, how these principles operate in practice, especially across communities that differ politically, socially and culturally, are heavily debated [5]. Though there may not be differences in standards of medical ethics across sociocultural contexts [6], competing ethical principles may influence research practices and implementation. For example, controversies surrounding the use of “standard of care” were highlighted in several well-publicized studies of mother-to-child HIV transmission in the late 1990s because of the use of placebo-control groups [4,7,8]. Important ongoing ethical considerations in international research include the ability of participants and communities in resource-limited settings to benefit from research [9,10] and appropriate informed consent processes [4,11].

Several factors may increase the vulnerability of individuals and communities participating in research, including, lower socioeconomic status, less experience with and understanding of biomedical research, and poor availability and accessibility of health services [12]. Because these vulnerabilities may be particularly prominent in resource-limited communities, additional mechanisms for ensuring adequate protection for research participants should be considered [4]. The need to apply and uphold standard ethical frameworks across significantly different communities and within vulnerable populations has led to recommendations stressing the role for community engagement as part of the ethical research process [13,14]. Involving communities in aspects of research activities like protocol development and research conduct can lead to greater protections for communities and individual participants [15,16], but little empirical data inform the best practices and models of community engagement for research in sub-Saharan Africa [17,18].

Informed consent is a fundamental ethical principle of biomedical research to protect vulnerable populations and minimize risks [19]; however, the optimal implementation of informed consent requires consideration of multiple factors. The type of scientific trial, particularly whether randomization is done and whether it is done in clusters or by individual, may shape which consent processes best protect vulnerable subjects [20]. Moreover, in certain settings, individuals may be less likely to understand the research being conducted or their rights as participants [21,22], leading to concerns about the adequacy of standard informed consent practices. Illiteracy, lack of formal education and differing views of health and research complicate informed consent in all settings and may be particularly significant in resource-limited settings [21,23]. Both decisions favoring participation and refusals to participate in research may result from a lack of information or appropriate explanations, including perceptions of access to benefits such as healthcare [24]. When a community has limited access to health services, research may be used as or considered a means to access healthcare [25,26]. While conflating research and healthcare is often referred to as the “therapeutic misconception”, this may not be a misconception in settings where the research is the only access point to services [27,28]. Finally, how the participants conceptualize individual autonomy and whether community or social autonomy supersedes the individual can have important implications for informed consent processes [29,30]. The balance between individual autonomy and social or political choices may significantly influence the implementation of research [31]. A qualitative study of researchers in developing countries emphasized the need to allow more operational flexibility in the informed consent process to account for varying community contexts [32]. Furthermore, a recent review of research on standard informed consent suggests greater community involvement may be needed to better protect participants in all settings, particularly resource-limited settings [33].

International research bodies recognize the need to increase community engagement in research activities [34,35]. How a given community understands research and how to best engage communities in the process of consent are still open questions in many international research settings [36]. A literature review related to informed consent found an absence of data from the developing world, and particularly from sub-Saharan Africa, on how communities’ perceptions of informed consent related to the experiences of participation in the research [11]. Moreover, the limited empirical research on the informed consent process in resource-limited settings suggests community perspectives require further investigation within multinational research settings [21,25,37-40]. A needs assessment of research ethics issues among a partnership in western Kenya found adapting informed consent processes to involve community consent was considered a major challenge [41].

As multinational research collaborations seek to answer these questions about how best to conduct informed consent and to increase community engagement, existing community structures for dialogue and decision-making may offer important venues for consideration. In many Kenyan cultures, traditional community assemblies called *mabaraza* are used for sharing information and for gathering community opinions on issues [42]. These *mabaraza* have the potential to serve a role in research investigation and development, as well as in the consent process. For the purposes of understanding community perspectives or beliefs, a *baraza* (singular form of *mabaraza*) can be used as a venue for qualitative investigation, and because the *baraza* holds cultural significance, it may produce more meaningful community involvement than researcher-organized boards or focus groups. A recent study investigated the use of *mabaraza* to understand community perspectives on the USAID-Academic Model Providing Access to Healthcare (AMPATH) HIV care program in western Kenya and found the forums were useful in eliciting community perspectives and understandings of health programs [42]. A qualitative case study in northern Ghana investigated the use of *durbars*, traditional, formal community gatherings that include cultural activities, as a form of community engagement with a biomedical research project [43]. They found that engaging the community within this existing cultural forum was effective in gaining community perspectives on various aspects of research and limited social disruption.

As a group conducting collaborative research within the context of a long-standing partnership between a North American medical school and a Kenyan medical school [44-46], we sought to use *mabaraza* to understand the community’s perspective on research and informed consent processes in western Kenya. Our goal was to inform ethical research practices in this setting and in similar resource-limited settings. Using rigorous qualitative research methods, this study sought to evaluate the community’s perspectives on research, informed consent, and use of the *baraza* within the research process to engage families in western Kenya.

## Methods

### Study design using *mabaraza*

We conducted a qualitative research study using dedicated *mabaraza* as a forum for engagement with community members about their perspectives on research and informed consent in western Kenya. The *mabaraza* were officially organized by a Chief or sub-Chief, with significant involvement of village elders in the dissemination of information about the *mabaraza* to villagers to ensure an open invitation for community members to attend the gathering. The *mabaraza* were employed similarly to how focus group discussions would typically be used for qualitative inquiry; these community assemblies were conducted specifically for eliciting group discussions and interactions about the community perspectives on this particular content area [42]. The *mabaraza* differ from focus groups, though, in that they are typically much larger, they include a more heterogeneous population, and despite having a facilitator and a semi-structured interview guide, they are more open discussions that yield output following the group’s reflections and experiences [42]. While community leaders or elders typically organize *mabaraza*, the discussions are usually multidirectional and led by participants rather than a facilitator. Use of the *baraza* was chosen specifically because it can yield more spontaneous and diverse individual and community perspectives [42], as well as because of its potential to capture the perspectives of community members in a specific administrative location in which ongoing research was underway.

### Setting

The study was conducted in the Uasin Gishu county of western Kenya under the auspices of the AMPATH Research Network. Uasin Gishu is located in the Rift Valley province and constitutes 3 constituencies (Eldoret North, Eldoret East and Eldoret South). (https://opendata.go.ke) Uasin Gishu county has a population of 894,179 people, of whom 38.6% live in an urban setting. Almost half of the population is estimated to live below the Kenya poverty line of 1,562 Kenyan shillings ($18.75 USD) per month in rural areas; and 2,913 Kenyan shillings ($34.97 USD) in urban areas per person per month.

Indiana University School of Medicine and Moi University School of Medicine have worked in a collaborative partnership for education, research, and clinical care in western Kenya since 1989 [46]. In 2001, Indiana University School of Medicine, Moi University School of Medicine, and Moi Teaching and Referral Hospital partnered to create a model HIV care system in western Kenya, now called the USAID-Academic Model Providing Access to Healthcare (AMPATH) [44,45]. AMPATH has enrolled over 140,000 patients in western Kenya, and currently follows approximately 75,000 active patients (including over 24,500 children) at 65 health facilities in three provinces of western Kenya. AMPATH provides primary health care services and access to free antiretroviral treatment (ART), as well as comprehensive nutrition services, psychosocial support, and economic development training.

AMPATH has a highly functioning research network with shared North American and Kenyan leadership. All research endeavors follow a collaborative partnership pattern, with a focus on improving health for children and adults in Kenya. AMPATH currently has 109 IRB-approved research protocols underway. Ethical review is conducted by an OHRP-approved Institutional Research and Ethics Committee.

### Study population

This study was conducted within the auspices of an ongoing longitudinal research project intended to improve the health and well-being of orphaned children by evaluating the potential effects of their care environment on their physical health and psychosocial well-being. (1R01HD060478-01A1). The parent study uses standardized site assessments, medical examinations, and psychosocial assessments to follow approximately 3,000 orphaned and separated children in the Uasin Gishu county of western Kenya for 5 years.

Aiming to assess the community perspective on biomedical and behavioral research and informed consent processes in this county, two *mabaraza* were called in strategic peri-urban locations in the district in which the parent study is taking place (Pioneer and Kapsoya Locations). The chiefs in these locations chose appropriate dates and then assistant chiefs invited the target communities to attend the *mabaraza* through the village elders. Participation was open to any community members who wanted to attend; however, the Chief and Assistant Chief specifically asked the village elders to each invite at least one caregiver of orphaned and separated children to attend, in addition to members of the general public. This purposeful invitation was extended because of the project’s particular interest in the involvement of vulnerable children in research in this setting. The *mabaraza* participants consisted of the provincial administration (the Chief, Assistant Chiefs, the District Children’s Officer and village elders), caregivers of orphaned and separated children and members of the general public, both male and female, including elderly caregivers.

### Procedures

*Mabaraza* in the Kapsoya Location and in the Pioneer Location of the Uasin Gishu county of western Kenya were called by usual community standards under the coordination of the chiefs and assistant chiefs. The *mabaraza* were held in enclosed, large meeting rooms, one the municipal council hall and the other at the locational Chiefs’ camp. The *mabaraza* were conducted in June and July of 2011 in Kiswahili by a trained facilitator. A prepared interview guide, containing open-ended qualitative questions, was used to solicit responses during a 3-hour session for the *mabaraza*. [Interview guides available from the corresponding author upon request.] Questions were based upon review of the literature and the input of local health care providers and specialists in research ethics and *mabaraza* function. The final questions covered multiple areas related to the experience of community-based research and consent, as well as issues related to involving vulnerable children in research. The participants granted permission for audio-recording of the sessions to allow for later transcription. Field notes were taken during and immediately after the encounters. All of the recordings were transcribed and then translated into English by a trained translator. The study procedures were approved by the Institutional Review Board of Indiana University in Indianapolis, Indiana and by the Institutional Research and Ethics Committee of Moi University School of Medicine in Eldoret, Kenya.

### Analyses

A system of manual, progressive coding of the transcripts was used to identify emerging central concepts [47]. The initial stage of constant comparative analyses was done through open coding by two independent investigators (Vreeman and Kamaara), involving a line-by-line analysis of each transcribed page of informant data to elucidate meanings and processes. These independent analysts also extracted and compared themes. Before condensing the codes, the analysts read the data several times, including comparison of a final review of all open codes from each of the analysts, followed by re-coding based on consensus by three analysts (Vreeman, Kamaara, Scanlon). Hypotheses and concepts were developed inductively from the data. Finally, relationships among the codes were integrated and refined. Selected quotations were used to illustrate key themes within the conceptual model.

We incorporated triangulation or verification on several levels. First, we analyzed and compared transcript data from *mabaraza* in two locations, as well as field notes. Second, independent reading, coding, comparison, and summary of themes were performed by three investigators (Vreeman, Kamaara, Scanlon). Finally, we incorporated two sources of peer debriefing and peer checking of transcripts and themes (Braitstein, Kamanda).

## Results

We collected data from 108 participants at the 2 *mabaraza* (79 male and 29 female). The Kapsoya *baraza* consisted of 37 participants, of which 17 were female and 20 male, including one chief, 11 village elders, and 25 caregivers or other community members. The Pioneer *baraza* consisted of 71 participants, 12 female and 59 male, including one assistant chief, 35 village elders, and 35 caregivers or community members.

### Knowledge and attitudes towards research

Community members generally understood research as a form of inquiry. They frequently defined research as searching for “*the cause of a problem*” or seeking to understand how something worked. As two community members from different locations shared: *“establishing the cause of the problem is research”* and *“research is the search for the root cause of something”.*

Although research was viewed as an endeavor to gain understanding or knowledge, research was typically seen as something that would directly benefit the community. This became a pervasive theme throughout the discussion of informed consent; and even at the level of defining research, participants expressed expectations for research benefits such as:

"“The information from the research should be useful for the overall growth of the community…The community’s well-being will be the reason as to why the research has to be carried out.”"

While these benefits were sometimes characterized as advances in growth or well-being, other concrete benefits described included new water sources, medicines, and tuition for children.

Participants thought that the community’s attitude towards a research study would be shaped primarily by the goals and the benefits of proposed research. When questioned about what the community would want to know about a research project before it would be considered acceptable, they focused on wanting to understand the reason for the research and to know the benefits of the research for the community before making decisions about acceptability. Participants emphasized their desire to understand what would take place and why. They seldom verbalized wanting to know about the potential risks involved with research, instead stressing the goals, procedures, and benefits. As one participant expressed:

"“There is no way I will accept to participate in research before knowing why they want to carry it out. I must know why they want to do the research and what it is all about.”"

The participants emphasized that the *“goal of research is very important”,* but at the same time, *“we need to know the benefits.”* The expectation that benefits would accompany the research and the need to know what those benefits would be were emphasized. *“Once we know the goal of the research, then next we need to know the benefits of the research,”* explained one participant. Both the information gathered through the research and additional direct benefits were considered to “*assist the community*”, and the indirect and direct benefits were at times conflated. For example, when asked about what the information was that would assist the community, a community member’s first response was:

"“An example is something that can assist the community. For example, during the period of farming, the community can be given seed and fertilizer. In times of sickness, they can be given drugs.”"

Research was also widely assumed to involve HIV-related investigations, even though neither HIV nor the AMPATH treatment program were mentioned in scripted questions. The participants assumed that any testing done within the context of a research study would include HIV testing, and the risks of HIV disclosure and subsequent stigma were viewed as major risks to research participation. As one woman described:

"“When research is carried out, worries have to be there. Why? This is because they are coming to my house and all my secrets will be revealed to my husband and children. The research must be handled with utmost confidentiality and care.”"

She specified that these “*secrets*” causing worry would be related to HIV infection. Participants greatly feared having HIV diagnosed and then disclosed to other people since this might result in stigma and discrimination. The impact of having children diagnosed with HIV was also a concern when discussing the participation of children in research studies. When participants were asked about their worries about research in their community, they most commonly described worries related to being diagnosed with HIV such as *“bad results caus[ing] a lot of worry*” or “*worrying about finding disease.”* The concerns about research were focused on the negative information with which they would need to cope, rather than harms that might result from the research itself. Many statements about worry related to research, such as the research being “*about death and research that will yield bad results*”, were in reference to research about HIV. In addition to HIV testing itself, the participants worried that the presence of researchers, particularly within one’s home as an unidentified visitor, might be interpreted by others as a sign that the family had HIV.

In addition to the fears about testing positive for a disease or dealing with the negative implications of having HIV infection, participants suggested that research could cause worry if there were not clear benefits for the community. Again, the strong expectation in this setting was that research would result in benefits to the community, and particularly to impoverished community members. Participating in research was considered to be a waste of time when there was not a direct benefit:

"“[A worry about research] is wastage of time – waiting for research and then having no assistance given to community.”"

"“The community needs assistance, but if no assistance is given by the research team, then it will be in vain for the community. For instance, the Kapsoya community has a water shortage, and they are promised by the research that an engineer will drill a water borehole for them or bring tapped water for the community. Later, they are told that the project has stalled. This will break people’s hearts, and next time, they will not accept to participate in any research because there are no benefits.”"

A minority of comments in the *mabaraza* discussed risks involved with other aspects of the research, such as if the research involved taking particular medicines which might cause harm to children or animals who got into them accidentally.

### Community understanding of informed consent

Among this population of Kenyan adults, the conceptualization of informed consent focused primarily on the extent to which the participants would want to understand the scope of the research. “*Understanding all about the research*” and “*information on the research*” were the primary responses as to what informed consent meant to them. Most of the terms participants used in translation for “consent”, including *“authorization*” and *“permission*”, reflected understanding that the consent process was one during which the participant would be asked to indicate their agreement to participate in the research. There was also general understanding that giving consent would mean one had committed to carrying out the research activities: *“It means you accept to be asked all questions and [*[38]*] participate.”* Many participants also recognized that informed consent would include learning about both the risks and benefits for the research. Receiving *“information on the benefits and risks*” and *“being informed about the benefits and risks of the study”* were specifically mentioned by *baraza* participants from both groups. In both groups, the focus was more on knowing what the benefits would be, but they also mentioned being aware of the risks.

Some participants did describe culturally unique components that they viewed as part of informed consent. These comments revolved around the community’s awareness of the research and expectations that a community-based contribution would be part of consent. For example, several participants described informed consent as a process of “*creating awareness*” in the community or a process of the community “*contributing*”, without discussing individual agreement for participation. One of the groups also discussed informed consent as *“a process of contributing. The people are given a chance to contribute.”* The participants thus conceptualized informed consent as a process by which the community would become more aware of the research and also in which the community’s own participation would be defined.

### Community consent for research

In these western Kenya locations, participants endorsed an increased role for the community in making decisions about research participation. The groups stressed the importance of bringing community members together for explanations of planned research and giving them opportunities to ask questions and to come to a group consensus about participation. As indicated by one participant, when considering consent for research, the community should be involved in order to *“bring [people] together, discuss the different issues, and come to an agreement.”*

While the general consensus was for the community to be brought together and to agree to the research, many participants conceptualized the process of community consent as being led and mediated by a community leader. In explaining the role of these community leaders, one participant explained, *“The village elders are the ‘eyes’ of the community.”* The participants suggested that the community must first choose *“a spokesman*” or “*those who represent the community, for example the chief’s office or churches*.” These representatives of the community were considered the ones a research group should approach about a particular project. The sequence was first to “*approach the village elder*” and then *“a baraza must be called…[where] the whole process will be explained to the community.”* Some participants did not feel that a group discussion would be mandated, but that the community could *“elect one leader… [who] gives consent for leading the rest.”* The chosen community leader could either be the organizer and leader of the discussions or the surrogate decision-maker for the community.

The participants emphasized that the community *“must have information that is easy and simple to understand.”* Bringing community members together in a gathering such as a *baraza* was the most frequently cited idea for disseminating information about a research project, although participants suggested that pamphlets and public announcements over the radio or over a public address system could also be used. As to the content of this information to be provided to the community, the participants emphasized that the community would need to know how the research would benefit them. Information on how the research “*will assist the community*”, “*will progress the community*” or “*benefit the community*” were the most common responses for what the community would want to know before a decision was made about research participation.

Community consent was seen generally as a supplement to individual informed consent, not as a replacement. While it was widely accepted that “*the community should be brought together”* to consider whether a research project was acceptable, individuals would still be expected to give their agreement. For pediatric participation in research, a broader role was envisioned for community members; not only parents and guardians, but also teachers, older siblings, pastors and children themselves were considered individuals who might consent for a child to participate in research. Community consent was considered a potential alternative to parental consent in cases where children were orphaned, *“times when the child is not well taken care of”*, when *“there is an outbreak or emergency”,* or when a parent *“doesn’t have the ability to raise the child”*. The acceptability of community consent in cases where the child was not otherwise being taken care of seemed to follow a common belief that *“the community is a family”.*

## Conclusions

An emerging priority in international health research is increasing the role of the community in all aspects of research activities [34,35]; however, models for community engagement in research in sub-Saharan Africa remain undefined. Examining community members’ understanding of research and informed consent within a multinational research collaboration in Kenya provides insight into how community engagement might be strengthened in similar settings. While community members generally understood the concepts of research and informed consent, their emphasis on the benefits reaped from research participation and their equating of research with HIV testing or care could inform education around topics such as research risks. Community-level consent was widely accepted as either a prerequisite to, a supplement to, or a substitute for individual informed consent in this setting.

Employing the *baraza* -- a culturally accepted public assembly for the purpose of community dialogue [42] -- we gathered novel data on community members’ understanding of research, informed consent, and community consent. We built on previous qualitative work in Kenya that used focus group discussions with community leaders and members to better understand their perceptions of research and informed consent [17,26], and we tried to fill the continued gaps in understanding how a community’s perspective on informed consent relates to participation in the research [11]. While the community’s understanding of research generally encompassed the principles of inquiry and investigation, the strong expectation to receive direct benefits for research participation was an important belief to consider and understand. The community members often focused on the goods, services, capacity building or development that might be offered as part of the research activities. This is consistent with other findings from poor communities, where research activities were equated with development projects whose aim is to improve conditions in the community, rather than to collect information or test interventions [25]. While some research studies offer access to health care or other resources of value to the participants, others might not benefit one’s health, education, or finances. In our work, the participants expressed that the potential benefits of the research would be scrutinized – rather than the protocol. These expectations for direct benefits should inform how researchers present research proposals to the community and how they protect vulnerable individuals within that community. This community view could shift the risk-benefit ratio such that research benefiting the community would be endorsed at the community-level despite individual risks to the participants. These findings also point to the need to be explicit about possible risks. In this context, studies with few direct benefits may be unlikely to recruit participants from similarly underserved or impoverished settings, while studies with obvious benefits might draw in participants despite having significant risks. Considering this interpretation of the risk/benefit ratio for this particular setting and striving to make clear the risks that the participants might not otherwise consider would be important targets for researchers within this cultural context. A multinational research team might be seen as even more likely to offer direct benefits for research participants, reinforcing the need for researchers to understand the unique community perspectives in order to adequately address community concerns and provide protections for vulnerable populations. Understanding how the history, poverty, and unique resources of a given community shape the community’s attitude towards research is vital for researchers entering that community.

Another important cultural perception revealed in the *mabaraza* was the conflation of HIV and research. Given that the most sophisticated healthcare system in this community in western Kenya was designed for HIV care and that much of the research which has taken place has been related to HIV, it is conceivable why participants would assume biomedical research involved HIV testing or HIV-related outcomes. AMPATH has conducted research in this setting since its inception in 2001, and AMPATH’s services within this county have included home-based HIV counseling and testing, and multiple locations provide voluntary counseling and testing for HIV. Conflating HIV and research may thus be a unique finding to a setting with significant HIV services. However, since HIV infection continues to be significantly stigmatized in this society, the equation of research with HIV means that HIV-related concerns for privacy and confidentiality are prominent in the minds of community members. In fact, “risks” associated with research were largely interpreted to mean risks related to diagnosis and disclosure of HIV status, rather than any other harm. Studies with no HIV-related components might want to take particular care to make this clear to potential participants. Issues of confidentiality, stigma, and HIV disclosure need to be addressed carefully within any research project in this setting.

Community consultation commonly refers to engaging communities and eliciting feedback from community members that researchers can incorporate into the design of their research work to enhance protection and benefits for the community, increase legitimacy and share responsibility of activities and outcomes [48]. Community advisory boards have been utilized for community consultation in HIV research [49,50], but there is little empirical data on the effectiveness of these boards in the consultation process [51]. Communities in western Kenya have worldviews that emphasize communitarian rather than individualistic living, a philosophy that may render community consultation all the more necessary [52]. The use of a traditional community assembly such as the *baraza* offers a potential venue for community consultation, and for additional research activities such as community consent. Involving the community in review of research proposals in assemblies such as *mabaraza* was widely perceived as culturally acceptable and an important protective measure. Community consent is a process of seeking permission from the community to solicit individuals to participate in research [48]. Our findings from Kenya suggest that this is a setting in which community consent may be appropriate and necessary, even before individual consent, in order to extend protections from the individual to the community-level [41,53]. Using community consent as a supplement to individual consent would be in accordance with the 2002 Council for International Organizations of Medical Sciences International Ethical Guidelines for Biomedical Research Involving Human Subjects, which endorses “obtaining permission from a community leader, a council of elders or another designated authority.” [35] Our findings begin to answer key questions needed to implement this guideline: 1) defining the community, 2) describing the content and purpose of community consent, and 3) identifying the legitimately empowered local leaders who could make decisions about research on the community’s behalf [54,55]. As there have been very few studies investigating community consent processes in sub-Saharan Africa [18,38,40] and no guidelines as to its implementation, these formative data offer an important description of how one community would answer these questions. The limited body of research investigating effective strategies for community engagement in consultation and consent processes warrants further investigation [36].

Community consent might offer a particular benefit to especially vulnerable populations, such as orphaned children, where the community’s consideration of the research could offer an additional level of protection against potential harms or the opportunity to advocate for including these children when benefits might be present. The potential advantages of community consent need to be examined carefully against potential harms that could result if the community were to value community benefits over the protection of an individual; however, the usual measures for protecting against abuse, from emphasizing researcher integrity to requiring local IRB review, could help minimize those risks. Not only must researchers ensure that the procedures, risks, and benefits to their proposed work are well understood, but they must take precautions to minimize risks for vulnerable individuals.

Dissenting views also merit further consideration when considering community consultation or consent. Balancing individual autonomy and the choices of the broader social context must be carefully weighed in implementing ethical research [31]. Child dissent was not discussed, nor was the possibility that the voices of some members of the community might not be heard in the context of community deliberations or assemblies. Within this setting in Kenya, women, younger community members, and families of lower socio-economic status in the village may not feel able to speak freely in group deliberations, and thus their opinions may go unheard in community discussions. To try to counter this tendency in our assemblies, the facilitator of the *mabaraza* specifically asked for responses from women within the groups and periodically asked participants who had been quiet to offer their opinions. This increased participation among groups that might face gender or social inequalities, and did result in hearing some diverse opinions. However, their concerns may not have been fully expressed, and addressing these inequalities within the context of a community assembly requires direct attention or may require complementing the community *mabaraza* with smaller, more homogeneous group discussions (men only, women only, youth only, etc.). A family or individual’s decision to withdraw from a study for which community consent has been given is another important form of dissent to address from the beginning. The participants in these discussions did not talk about that issue. The weight given to community consent compared to individual consent or dissent may also be altered by the design of the study, taking into account factors such as cluster or individual randomization and whether individual consent is possible. Additional investigation of how to address, protect, and respond to minority views within this cultural context would be helpful.

This study does have limitations that merit consideration. The model relies on the contextual data and lived experiences of subjects in a very particular part of the world – two locations in western Kenya. Thus, the results may not generalize to other geographic locations. The *mabaraza* took place in peri-urban areas that would be classified as slums, which may make the findings less generalizable to more rural or urban settings. Nonetheless, this is a population often under-represented in research, and the population caring for many of the orphans and vulnerable children we sought to evaluate. Furthermore, the methodology has the inherent limitations of using relatively small, convenience samples which can limit generalizability. However, the lived experiences of subjects in this particular resource-limited setting may have more generalizability for subjects in other resource-limited settings than do similar studies conducted in resource-rich settings. Despite these limits to the sample, validity was supported by thematic saturation. In addition, there have been no published studies specifically using *mabaraza*, or similar traditional assemblies, to gain community perspectives on informed consent processes in biomedical research settings, and so the data from this population offer an important window into ethical cross-cultural research practice. A *baraza* gathering might not lend itself to hearing from every participant or to collecting in-depth responses from any one participant in the manner of an individual interview or a smaller focus group. Nonetheless, it does provide a community overview and broader perspective on the community’s beliefs. Moreover, in this context, it made use of a structure being proposed to serve a similar purpose in future research endeavors and thus testing its own possibilities.

Our study suggests that any attempt to conduct ethical multinational health research must take into account the community’s understanding of research and informed consent, particularly when involving vulnerable populations. The informed consent process and the individual weighing of risks and benefits may not be well understood by a given community, and so these procedures need to be addressed in a manner that is accessible to all community members, regardless of their socioeconomic background. Researchers should also recognize that there may be strong expectations of direct benefits for research participation, as well as expectation that HIV testing or care are part of the activities, and researchers should incorporate education on these issues relevant to the particular project. The community’s beliefs about community consent can be examined, and in settings where consent given by a community leader is considered appropriate, these procedures may be incorporated into research development and implementation. Moreover, traditional assemblies within these cultures, such as a *baraza* in Kenya, can be used effectively to gather these critical cultural data and act as a forum to obtain community consultation or consent.

## Competing interests

The authors declare that they have no competing interests.

## Authors’ contributions

**RV** led the development, analysis, and writing of this manuscript. **EK** assisted in the conduct of the analysis of *baraza* data and contributed to the writing and revision of the manuscript. **AK** participated in the development of this project, facilitated each of the *baraza* assemblies, and participated in the writing and revision of this manuscript. **DA** participated in the development of this project and in the revision of this manuscript. **WN** contributed to the development of this analysis and the writing and revision of this manuscript. **LA** contributed to the development of this analysis and the writing and revision of this manuscript. **SA** contributed to the development of this project and the writing and revision of this manuscript. **PG** contributed to the development of this project and the revision of this manuscript. **MS** participated in the analysis of this work and contributed to the writing and revision of the manuscript. **PB** led the development of this project and contributed significantly to the writing and revisions of the manuscript. All authors read and approved the final manuscript.

## Authors’ information

**Rachel Vreeman** is Assistant Professor of Pediatrics at Indiana University School of Medicine and Co-Field Director for Pediatric Research for the Indiana University –Kenya Partnership and Academic Model Providing Access to Healthcare (AMPATH). Dr. Vreeman has extensive research experience in western Kenya that focuses on improving the provision of healthcare to children in resource-limited settings. She specializes in measuring and sustaining HIV adherence and evaluating disclosure issues in pediatric populations in resource-limited settings. As the co-chair of a multinational Kenya Pediatric Research Working Group that operates within AMPATH, she is particularly interested in promoting the ethical implementation of research within the vulnerable pediatric population in Kenya. She also serves on the curriculum and admissions committees for the IU-Moi Academic Research Ethics Partnership.

**Eunice Kamaara** is a professor in the Department of Philosophy and Religious Studies at Moi University in Eldoret, Kenya. She is also a student in the Masters in International Health Research Ethics program. Her research interests focus on informed consent in post-colonial contexts.

**Allan Kamanda** is the study coordinator for the Orphaned and Separated Children’s Assessments Related to their Health and Well-being (OSCAR) research study in western Kenya, which is following the outcomes of a large cohort of several thousand orphaned and separated children. His research interests focus on the consenting process among vulnerable populations such as orphaned children and street children.

**David Ayuku** is an associate professor in the Department of Behavioural Sciences, School of Medicine, Moi University College of Health Sciences in Eldoret, Kenya and Co-Principal Investigator for the Orphaned and Separated Children’s Assessments Related to their Health and Well-being (OSCAR) research study in western Kenya. His research interests focus on the issues of street children and orphans.

**Winstone Nyandiko** is an Associate Professor and the Head of the Department of Child Health and Paediatrics, School of Medicine, Moi University College of Health Sciences in Eldoret, Kenya and Associate Program Manager for the AMPATH partnership, serving as the Co-Director for the AMPATH Research Network. Dr. Nyandiko is also the Pediatrician-In-Charge for the AMPATH Pediatric HIV Care program. His expertise lies in evaluating the clinical management of HIV-exposed and HIV-infected children and implementing research within the Kenyan health care system.

**Lukoye Atwoli** is a Lecturer in the Department of Mental Health in the School of Medicine, Moi University College of Health Sciences in Eldoret, Kenya and secretary of the Kenya Psychiatric Association. His research interests are in psychotrauma in children and adults, general hospital psychiatry and the interface between HIV/AIDS and mental health.

**Samuel Ayaya** is a Professor in the Department of Child Health and Paediatrics, School of Medicine, Moi University College of Health Sciences in Eldoret, Kenya and Co-Chair of the Kenya Pediatric Research Working Group. His research interests include children’s nutrition, growth, and development, the progression of HIV in children, and child abuse and neglect.

**Peter Gisore** is a Lecturer in the Department of Child Health and Paediatrics, School of Medicine, Moi University College of Health Sciences in Eldoret, Kenya. His interests center around clinic and community-based research on maternal and newborn health.

**Michael Scanlon** is a Global Pediatric Clinical Research Scholar with Indiana University School of Medicine and the AMPATH partnership. He is focused on research to support long-term care of children with HIV in resource-limited settings, including disclosure of HIV status and adherence to HIV therapy, as well as community-based participatory research.

**Paula Braitstein** is Associate Research Professor of Medicine at Indiana University School of Medicine, Co-Field Director for Research for the Academic Model Providing Access to Healthcare (AMPATH), and Co-Principal Investigator for the Orphaned and Separated Children’s Assessments Related to their Health and Well-being (OSCAR) research study in western Kenya, which is funded by the National Institute of Child Health and Human Development (1R01HD060478-01A1). Her research expertise lies in evaluating care outcomes including for HIV-infected patients, orphans, separated children, and other vulnerable populations.

## Disclaimer

The views expressed in this article are those of the authors and do not necessarily represent the view of the Indiana University School of Medicine or the Moi University School of Medicine. The authors have no conflicts of interest to disclose. The first author had full access to all the data in the study and takes responsibility for the integrity of the data and the accuracy of the data analysis.

## Pre-publication history

The pre-publication history for this paper can be accessed here:

http://www.biomedcentral.com/1472-6939/13/23/prepub
